# Post‐prophylaxis *Toxoplasma* chorioretinitis following donor–recipient mismatched liver transplantation

**DOI:** 10.1111/tid.12589

**Published:** 2016-10-04

**Authors:** G.J. Webb, H. Shah, M.D. David, S. Tiew, N. Beare, G.M. Hirschfield

**Affiliations:** ^1^ National Institute of Health Research Birmingham Liver Biomedical Research UnitBirminghamUK; ^2^ St Paul's Eye UnitRoyal Liverpool University HospitalLiverpoolUK; ^3^ Clinical MicrobiologyQueen Elizabeth HospitalBirminghamUK

**Keywords:** *Toxoplasma gondii*, toxoplasmosis, ocular, chorioretinitis, liver transplantation, immunosuppression, disease transmission, infectious

## Abstract

Toxoplasmosis may be transferred by organ transplantation. The most common clinical presentation is with multisystem disease, although isolated ocular toxoplasmosis has been described. Many centers have suggested that universal use of co‐trimoxazole prophylaxis obviates the need for specific *Toxoplasma* testing. We report a case of donor‐acquired ocular toxoplasmosis after liver transplantation despite co‐trimoxazole prophylaxis. The diagnosis was confirmed by *Toxoplasma* polymerase chain reaction assay in conjunction with seroconversion. The fact that the infection was donor acquired was confirmed by serological mismatch and the absence of sporozoite‐specific antigen antibody in the recipient.

Ocular toxoplasmosis is a major cause of uveitis worldwide and may be particularly aggressive in the immunosuppressed 1. Transfer of the causative agent *Toxoplasma gondii* through transplantation has been reported for all solid organs, although infection most commonly presents with multisystem disease 2. Incidences vary between transplant programs, and it has been suggested that universal prophylaxis with co‐trimoxazole (TMP/SMX) is sufficient to obviate the need for *Toxoplasma* testing 3. Here, we report a case of donor‐acquired isolated ocular toxoplasmosis despite TMP/SMX prophylaxis, occurring 7 months after liver transplantation.

## Case report

A previously healthy 32‐year‐old British female patient presented with fulminant liver failure due to seronegative hepatitis. She received super‐urgent orthotopic liver transplantation from a brainstem dead UK donor. The recipient was seronegative for cytomegalovirus (CMV), but the donor was positive. The post‐transplant course was complicated by 2 episodes of acute rejection requiring high‐dose corticosteroids, in addition to standard immunosuppression with tacrolimus and mycophenolate. At discharge, liver biochemistry was normal. As per unit policy, she received 3 months of TMP/SMX and valganciclovir prophylaxis to prevent *Pneumocystis jirovecii* pneumonia and CMV infection, respectively.

**Figure 1:**
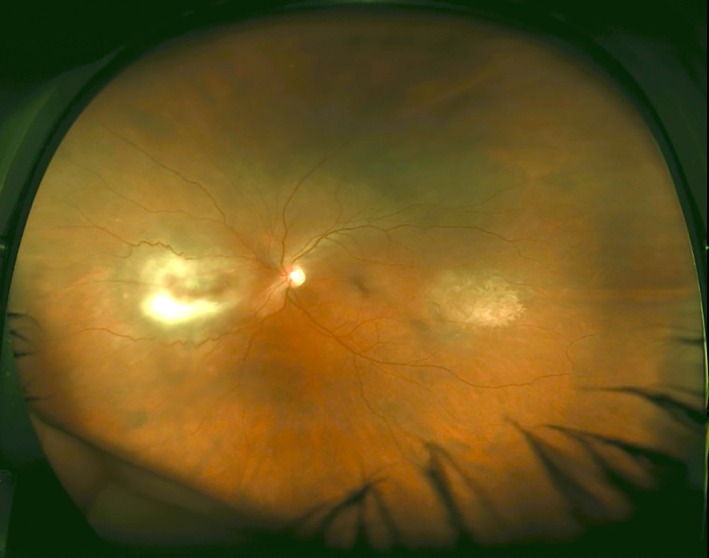
Wide‐field photograph of patient's left fundus, with eyelash artifacts inferiorly. Nasal region (left of the printed image) contains an active focus of chorioretinitis; the temporal region contains a region of chorioretinitis that has progressed to atrophy. Mild hazing caused by vitritis.

Seven months after transplantation, painless blurred vision affecting the left eye developed over several days. The patient had been well in the intervening period, with no other symptoms, and she was afebrile. External ocular examination was unremarkable. Fundoscopy of the left eye revealed 2 foci of chorioretinitis: an active nasal lesion and a temporal lesion that had largely progressed to chorioretinal atrophy. An overlying vitreous hazing was consistent with mild vitritis (Fig. 1).

As the differential diagnosis included *Toxoplasma* or CMV chorioretinitis, empirical therapy with ganciclovir and clindamycin was commenced initially. An aqueous humor sample revealed the presence of leukocytes, but routine microbiological cultures and polymerase chain reaction (PCR) for CMV and varicella zoster virus were negative.

*Toxoplasma gondii* DNA was detected by real‐time PCR amplification of a region of the RE gene (GenBank Accession no. AF146527). Serum *Toxoplasma* immunoglobulin (Ig)M was positive and IgG negative; IgG became weakly positive 20 days later. Retrospective testing of stored pre‐transplant recipient serum showed absence of anti‐*Toxoplasma* IgG and IgM, whereas donor serology was *Toxoplasma* IgG positive.

Ganciclovir was discontinued, the patient completed a course of clindamycin, and her vision normalized over several weeks. Subsequently, testing for a sporozoite‐specific antibody, according to the method described by Hill et al. 4, was negative (anti‐TgERP O.D. 0.198; positive cutoff >0.4).

Long‐term TMP/SMX secondary prophylaxis was commenced to prevent future recurrences, although was subsequently withdrawn because of myelosuppression. The patient went on to have a successful pregnancy while taking only tacrolimus and prednisolone immunosuppression.

On review of possible risk factors for acquiring toxoplasmosis, it was established that the patient had been given and followed routine advice to wash vegetables and to cook meat thoroughly. She had contact with her mother's healthy adult cat, but did not provide care for it.

## Discussion

This is the first reported case, to our knowledge, of *Toxoplasma* chorioretinitis occurring after liver transplantation despite TMP/SMX prophylaxis. Donor–recipient *Toxoplasma* serological mismatch (seropositive donor to seronegative recipients; D+R−), the onset of symptoms occurring 4 months after discontinuing prophylaxis, the absence of sporozoite‐specific antibody, and the lack of other obvious routes of acquisition suggest that our case could represent donor‐acquired infection. Of particular interest in suggesting the route of acquisition is the negative sporozoite‐specific antibody. In contrast, in a large US series of congenital toxoplasmosis, positive results of this assay suggested that the majority of such cases represent oocyst ingestion, even in the absence of risk behaviors 5.

*Toxoplasma* donor–recipient mismatch was a significant risk factor for post solid organ transplant toxoplasmosis across multiple solid organ types in a case–control series 6. The rate of infection appeared to be highest with heart transplantation, and this has been attributed to the *Toxoplasma* bradyzoite cysts' predilection for muscle tissue including myocardium 7. Furthermore, reports of separate recipients developing toxoplasmosis from kidney grafts from the same donor suggest that transferred infection can occur 6, 8.

The incidence of new *Toxoplasma* infection in the UK population is 0.25–0.5% per year, while toxoplasmosis of any presentation in donor–recipient mismatch has been reported at up to 40% across all solid organs 6, 9. Given that the cat in this case was adult and healthy, that living with fewer than 3 kittens is not a significant risk factor for acquisition of *Toxoplasma*, that other family members remained well, and that serum sporozoite‐specific antibody was negative, we believe feline contact to be an unlikely route of infection in this case 10.

A November 2015 Medline/PubMed search for “toxoplasmosis” and “transplantation” returned 506 abstracts, with 3 previous cases of possible donor‐derived ocular toxoplasmosis following orthotopic liver transplantation 11, 12, 13, 14. None received prophylaxis (Table 1). Our patient presented 7 months after transplantation and 4 months after cessation of prophylaxis. This is in contrast to a median of 31 days after transplantation for a series of all presentations of toxoplasmosis in liver transplant recipients 6 and 86.5 days among all solid organ transplants 12; however, details of prophylaxis are incomplete in the former study. The reported onset of isolated *Toxoplasma* chorioretinitis ranges from 21 days 11 to 9 months 6, and toxoplasmosis among all solid organ transplants is reported to be delayed in its presentation by prophylaxis (476.28 ± 415.70 days with prophylaxis vs. 48.81 ± 28.93 without), with no patients presenting while on prophylaxis 15. We hypothesize that our case's presentation may have been altered by prophylaxis and/or corticosteroids given for rejection.

**Table 1:** Reported cases of *Toxoplasma* chorioretinitis after liver transplantation

First author (reference)	Recipient age, gender; indication	Location	Immunosuppression	Diagnosis	Prophylaxis	Delay from transplant to onset	Therapy	Outcome
Singer 11	48, female; hepatitis C	USA	Aza, CsA	Pathological	None	8 months; 3 weeks after cataract surgery	None	Eye enucleated
Chiquet 12, 13	43, female; fulminant liver failure, possibly drug‐induced	France	Pred, CsA	Fundoscopic findings, serum anti‐*Toxoplasma* IgM	None	3 weeks	Pyri, Sulfad, Fol	No relapse for 1 year
Galván Ramírez 14	7, male; glycogen storage disease type IV	Spain	Pred, CsA	Fundoscopic findings, serum anti‐IgM and IgG	None	5 months	Pyri, Sulfad, Fol	No relapse 1 year; subsequent CMV retinitis
Webb (Present case)	32, female; fulminant seronegative hepatitis	United Kingdom	Pred, Tac, MMF	Fundoscopic findings, serum anti‐*Toxoplasma* IgM; PCR of aqueous humor	3 months TMP/SMX	7 months	Clind	No relapse 2 years

AZA, azathioprine; CsA, cyclosporine; Pred, prednis(ol)one; IgM, immunoglobulin M; Pyri, pyrimethamine; Sulfad, sulfadiazine; Fol, folinic acid; IgG, immunoglobulin G; CMV, cytomegalovirus; Tac, tacrolimus; MMF, mycophenolate mofetil; PCR, polymerase chain reaction assay; TMP/SMX, trimethoprim/sulfamethoxazole; Clind, clindamycin.

In this case, chorioretinitis was multifocal and vitritis was mild. These findings are consistent with those seen in human immunodeficiency virus/acquired immunodeficiency syndrome (HIV/AIDS) ocular toxoplasmosis patients 16. This presentation is in contrast to the immunocompetent host, where chorioretinitis is usually unifocal, may be accompanied by scars from previous resolved episodes, and may have severe overlying vitritis.

Laboratory diagnosis of toxoplasmosis traditionally relies on the presence of IgM antibodies or on IgG seroconversion. However, in immunocompromised patients, seroconversion may be delayed and even absent. The specificity and sensitivity of PCR is, therefore, particularly useful 3.

Primary prophylaxis for the prevention of toxoplasmosis is used by many heart transplant centers. Some advocate indefinite prophylaxis in donor–recipient mismatch. For other solid organ transplant programs, especially in the presence of a low background rate of *Toxoplasma* IgG seropositivity in the population, and where TMP/SMX *Pneumocystis jirovecii* prophylaxis is routine, donors and recipients are not routinely tested for the presence of evidence of past *Toxoplasma* infection 15.

Little experience has been published on the need for secondary prophylaxis after completing a treatment course for a confirmed *Toxoplasma* infection in the context of immunosuppression outside the HIV/AIDS population 17. Further studies are warranted given the side effect profile and costs associated with agents such as TMP/SMX; regular monitoring by blood PCR may be an alternative strategy.

In summary, we present a case of toxoplasmosis chorioretinitis in a liver transplant recipient. This is the first such case, to our knowledge, reported after prophylaxis, and we believe that it is likely to represent graft transmission. The onset months after transplantation, the relative rarity of the condition, and its atypical presentation may delay diagnosis in similar cases. We note the utility of parallel donor–recipient serology and of PCR analysis of aqueous fluid. *Toxoplasma* infection is still a potential risk for the liver transplant population despite widespread use of TMP/SMX prophylaxis.
